# Intra-Organizational Social Capital and Product Innovation: The Mediating Role of Realized Absorptive Capacity

**DOI:** 10.3389/fpsyg.2020.624189

**Published:** 2021-01-13

**Authors:** Beatriz Ortiz, Mario J. Donate, Fátima Guadamillas

**Affiliations:** ^1^Department of Business Administration, University of Castilla-La Mancha, Toledo, Spain; ^2^Department of Business Administration, University of Castilla-La Mancha, Ciudad Real, Spain

**Keywords:** intra-organizational social capital, realized absorptive capacity, product innovation, biotechnology and pharma industries, mediating role

## Abstract

This paper examines the influence on product innovation of factors based on a company’s transformation and exploitation of knowledge gathered from its intra-organizational relationships. Specifically, this paper analyses the influence of intra-organizational social capital (SC) (i.e., comprised of structural, relational, and cognitive dimensions) on realized absorptive capacity (RACAP). Moreover, it analyses the mediating role of RACAP on the relationship between internal SC and product innovation. Based on a sample of companies from the Spanish biotechnological and pharmaceutical industries, two hypotheses were tested using a structural equations model and the partial least squares (PLS) technique. The results support both hypotheses, suggesting that the development of strong and tightly knit links based on a common understanding and trust among company members lead the firm to develop dynamic capabilities for transforming and exploiting knowledge acquired externally, which fosters innovation based on new product development. Research limitations, implications and future research are also discussed by the authors of the paper.

## Introduction

Social capital (SC) has become recognized as a powerful factor to explain success in a large number of areas that concern organizational researchers (Cuevas et al., 2014). Organizations can develop new knowledge and improve their performance through company links with other agents (Maurer et al., 2011). Moreover, a firm’s network structure, along with high levels of engagement, cohesion, trust (Adler and Kwon, 2002; Mu et al., 2008) and a common vision (Doh and Acs, 2010; Alarcón et al., 2014), can help firms to detect innovation opportunities and be able to adapt to changes in the environment (Adler and Kwon, 2002).

Relationships within a company (intra-organizational or internal SC) are also an important driver for improving innovation procedures and promoting new ways to create value (Moran and Ghoshal, 1996). Intra-organizational SC could be defined as the organizational networks (Putman, 1993), trust, norms, mutual objectives and cooperation that exist between organization members (Fukuyama, 1995, 2001). It is an intangible asset based on the knowledge arising from the interaction of company employees (Adler and Kwon, 2002; Ben Hador, 2016; Ben Hador and Klein, 2019).

Several studies have shown the key role of absorptive capacity in innovation (see e.g., Fosfuri and Tribó, 2008; Cepeda et al., 2012; Leal-Rodríguez et al., 2014; Xie et al., 2018; Limaj and Bernroider, 2019). Absorptive capacity can be defined as a dynamic capability that allows companies to acquire and assimilate external knowledge [potential absorptive capacity (PACAP)], which has to be internally transformed and exploited [realized absorptive capacity (RACAP)] in order to create competitive advantages (Zahra and George, 2002). However, most of the existing research does not consider that each of these dimensions (PACAP and RACAP) could have different antecedents, which also condition the innovation process (Rodrigo-Alarcón et al., 2020). This paper suggests that literature on knowledge management (KM) should consider distinctly the analysis of knowledge transformation and application that comes from their intra-organizational relationship, and the abilities that the company must develop to absorb and exploit knowledge for innovative purposes (Ebers and Maurer, 2014).

Recent studies have shown, from a qualitative point of view, how internal SC positively impacts performance in knowledge-intensive contexts (Salas−Vallina et al., 2020). Other papers have analyzed the relationships between SC and PACP (knowledge identification capability and external knowledge acquisition) (Ortiz et al., 2017, 2018), and others have focused on examining the mediating role of absorptive capacity (PACAP and RACAP) on the relationship of SC and innovation (Duodu and Rowlinson, 2019; Wang et al., 2020). However, most of them have focused on analyzing SC from an external or inter-organizational point of view. We consider that a deeper quantitative analysis of how internal or intra-organizational SC contribute to the creation of new knowledge on the part of company members is important in order to understand how such knowledge can decisively improve company performance and, in particular, produce innovation results. In this regard, a contributing idea from this paper is that the company must develop the ability to assimilate and integrate the new knowledge of employees, coming from its intra-organizational SC, into its common knowledge base in order to improve its innovation capabilities.

Similarly, researchers have thoroughly explored the impact of intra-organizational SC on different innovation performance measures, but the empirical results are not conclusive about the nature of such a relationship. In that sense, there are authors that find positive, negative and even inverted U-shaped effects of internal SC on innovation. Different reasons can explain such divergent results. First, SC provides opportunities to an organization’s members to acquire new knowledge, but its impact on organizational performance depends on the way this knowledge is assimilated and used by the firm. Different kinds of knowledge (tacit, explicit) can also have different potential to impact on innovation performance.

We consider that one of the main reasons for these findings is based on the fact that research on this matter has not taken sufficient account of the mediating role that capabilities related to knowledge transformation and exploitation might have on the relationship between intra-organizational SC and innovation. Therefore, in order to bridge this gap, we propose in this paper that product innovation capabilities are strongly affected by the RACAP mediating effect, with internal SC being the main antecedent. This paper contributes to the growing research field on SC and its effects on product innovation through seeking a deeper understanding of intra-organizational SC assessment for creating organizational value. It could also be helpful to clarify the role of internal SC as an antecedent of RACAP, which has been as yet unexplored (Ebers and Maurer, 2014). The need to specify individually the antecedents of each dimension of the absorptive capacity construct has been justified empirically (Jansen et al., 2005; Ojo et al., 2017). In this sense, our literature review finds evidence about how resources based on a mutual common understanding, trust and strong links between employees foster the development of abilities for transforming and exploiting knowledge.

The structure of the paper is as follows. First, the conceptual aspects and research hypotheses are developed. Second, the sample and the research methodology are described. Next, the statistical testing of the hypotheses in a sample of Spanish companies in the biotechnological industry is analyzed. Finally, we present the main conclusions, limitations and future research lines which could improve our understanding of the influence of intra-organizational SC on innovation capabilities and the role of RACAP.

## Conceptual Framework

Social capital is “the sum of the actual and potential resources embedded within, available through, and derived from the network of relationships possessed by an individual or social unit” (Nahapiet and Ghoshal, 1998, p. 243). This research paper considers this approach as being the most comprehensive in explaining the SC construct for two reasons: (1) It allows SC to be integrated as a multidimensional construct according to the value of exchanged resources and capabilities among agents in a network; and (2) it makes it easier to analyze a company’s relationships from both inside (intra-organizational SC) and outside (inter-organizational SC) organizational borders.

In addition, Nahapiet and Ghoshal’s dimensional differentiation – *structural, relational and cognitive* – is used extensively by academics in the SC field (Zheng, 2010; Hsu and Hung, 2013). The *structural dimension* is characterized by all the interactive aspects present in the relationships between network members (Tsai and Ghoshal, 1998). Those elements are related to the network’s density and stability over time to both the greater and weaker strength of the connection between agents and their frequency and closeness (Inkpen and Tsang, 2005). Moreover, the *relational dimension* relates to assets, such as trust or reliability, which come from the relation and interaction between network members. In this dimension, the positive interactions between individuals or organizations over the years are included as sources of SC (Lesser, 2000). Lastly, the *cognitive dimension* describes shared codes that improve the mutual understanding of aims and behaviors within a social system (Tsai and Ghoshal, 1998; Blasco et al., 2010). The main aspects that define this dimension are common goals and a shared culture. These dimensions are intrinsically interlinked, and their joint analysis thus is crucial for a better understanding of how to exploit knowledge gained from a firm’s relations and to explain innovation performance (Martínez et al., 2012).

According to SC literature, innovation is the result of the connections, interdependences and exchanges of knowledge between a variety of agents in different circumstances (Landry et al., 2002). Thus, the influence of SC on knowledge creation and innovation has been extensively discussed in a number of academic papers (Nahapiet and Ghoshal, 1998; Gargiulo and Benassi, 2000; Moran, 2005; Chen et al., 2008; Zheng, 2010; Martín et al., 2011; Sánchez-Famoso et al., 2017; Ben Hador and Klein, 2019, among others). In that sense, different researchers have confirmed the impact of internal (intra) SC on innovation. However, we do not find conclusive empirical results regarding the nature of the connection between this kind of SC and innovation or how this connection works.

Some authors state that the ability to access and mobilize resources through internal relations is a key factor for improving innovation results (see e.g., Moran, 2005; Casanueva and Gallego, 2010; Delgado et al., 2011; Gu et al., 2013; Yan and Guan, 2018, Yeşil and Doğan, 2019). Another branch of research shows a negative relationship (see e.g., Gargiulo and Benassi, 2000; Edelman et al., 2004; Fleming et al., 2007; Sánchez-Famoso et al., 2017), explained by the fact that cohesive networks cause organizational inertia, provoke resistance to change and reduce the dissemination of new ideas. Finally, other studies reveal an inverted U-shape (see e.g., Leenders et al., 2003; Shi and Guan, 2016; Wang et al., 2017), explaining that both low and high levels of internal interaction hinder the development of creativity and innovation. In order to fill this gap, this research considers that the ability of companies to benefit from knowledge that arises from internal interactions is crucial in determining their strategic potential for creating competitive advantage related to innovation. Specifically, we propose that knowledge transformation and exploitation abilities (RACAP) can be helpful to explain the positive effect of internal SC on product innovation^1^.

Knowledge management literature points out how an effective knowledge absorption process enables a firm to improve its capabilities for dealing with changing environments and to be innovative and competitive (Cohen and Levinthal, 1989; Zahra and George, 2002; Todorova and Durisin, 2007; Escribano et al., 2009; Jiménez-Barrionuevo et al., 2011). According to Zahra and George (2002), absorptive capacity encompasses a set of organizational routines and strategic processes through which firms acquire and assimilate (PACAP), transform and apply (RACAP) knowledge with the aim of creating dynamic organizational capability. We consider that especially the internal abilities related to combining new and existing knowledge as well as the capabilities for improving, expanding and exploiting these combinations (Zahra and George, 2002) can encourage intra-organizational social interaction and resource exchange, which in turn will create new knowledge and ideas which foster greater product innovation.

On the one hand, as RACAP processes are internally developed (Cohen and Levinthal, 1989), it is typically considered that structural, relational and cognitive aspects of intra-organizational SC could have an important influence on them. In this respect, Ebers and Maurer (2014) asserted that when a company’s members have strong links, a common understanding about task development and mutual trust, its abilities to transform and use knowledge improves. Likewise, Selivanovskikh et al. (2020) stressed that high intensity of interaction between company members and social embeddedness encourages cooperation, communication as well as trustworthy and reliable behavior, all of which enable knowledge assimilation and exploitation. Strong ties provide a company with rich communication channels through which its members can exchange valuable knowledge that can be adapted and developed for new purposes (Levin and Cross, 2004; Smith et al., 2005). Similarly, close interaction facilitates knowledge mobilization and feedback loops, helping company members to understand knowledge obtained from others (Leonard-Barton and Sinha, 1993), and fosters joint problem resolution (McEvily and Marcus, 2005). In that sense, Upadhyayula and Kumar (2004) found that strong links developed in working environments are very important for employees when they seek advice regarding how to carry out specific tasks and procedures.

Moreover, trust between organization members increases the likelihood of new individual knowledge integration within a company knowledge base by means of its transformation, thus creating collective organizational knowledge (Tsai and Ghoshal, 1998; Wu, 2008; Selivanovskikh et al., 2020). Strong organizational bonds and a sense of reciprocity can facilitate knowledge mobilization inside the company as organization members will be motivated to share knowledge and information with those whom they trust (Uzzi, 1999). Additionally, trust acts as a social control mechanism that has a positive influence on both the amount of mobilized knowledge and the efficiency of that mobilization (Dyer and Nobeoka, 2000; Lane et al., 2001; Molina and Martínez, 2010). If company members rely on each other, they will sense that their know-how is trustworthy and safe (Fischer et al., 2004; Schoorman et al., 2007), and the likelihood that they transform and use each other’s knowledge will be higher (Mayer et al., 1995). This avoids concerns about opportunistic behavior (Galán and Castro, 2004), reduces the cost of knowledge search and verification (Dyer and Chu, 2003) and increases the probability and the efficiency of its further use (Selivanovskikh et al., 2020).

Finally, cognitive SC appears as a key factor that affects knowledge assimilation, transformation and exploitation (Rodrigo-Alarcón et al., 2020). In that sense, common and clear goals foster mutual understanding and exchange of ideas (Chow and Chan, 2008), which create a feedback loop that allows agents to understand and apply knowledge in a new and creative manner (Leonard-Barton and Sinha, 1993). Similarly, organizational culture ensures the appropriate context for social interaction (Máynez et al., 2012), and then enhances successful organizational KM (De Long and Fahey, 2000; Donate and Guadamillas, 2010). A company’s culture builds organizational rules and beliefs that can foster knowledge creation, such as improving learning and knowledge use at a variety of organizational levels (Naqshbandi and Kamel, 2017). From a resource-based view, this organizational culture for social interaction is an intangible asset that offers rent appropriation potential as it is embedded in the company’s processes and management systems, which makes it highly specific (Barney, 1991). This specificity involves a link to a company’s idiosyncratic KM processes and learning trajectories, all of which can basically be considered as RACAP resources (Zahra and George, 2002).

Additionally, company culture can contribute to avoiding the development of undesired behaviors in companies, such as change resistance, and encourages those others that boost knowledge assimilation and application, such as pro-activity, creativity or flexibility. Therefore, it would be expected that intra-organizational SC has a positive influence on RACAP. Thus, we hypothesize the following:

H_1_: Intra-organizational SC is positively related to RACAP.

On the other hand, there are many empirical studies that show the importance of the absorptive capacity for the innovation process (e.g., Cohen and Levinthal, 1989; Fosfuri and Tribó, 2008; Murovec and Prodan, 2009; Cepeda et al., 2012; Leal-Rodríguez et al., 2014; Ferreras et al., 2015; Xie et al., 2018; Limaj and Bernroider, 2019). For example, authors such as Fosfuri and Tribó (2008) emphasize that PACAP is a necessary, but not sufficient, requirement for accomplishing competitive advantages based on innovation. Companies also need to develop their RACAP, for which knowledge flows becomes essential to create new ideas, know-how and products. Similarly, Cepeda et al. (2012) or Xie et al. (2018) confirm that a firm’s absorptive capacity has a positive impact on a firm’s innovation performance, as knowledge transformation and exploitation abilities are vital for producing more innovation outputs. However, very few research papers consider how each distinctive facet of a company’s entire absorptive capacity has a specific effect on innovation capabilities (e.g., Cepeda et al., 2012; Leal-Rodríguez et al., 2014; Xie et al., 2018).

Theoretically, RACAP, as a dynamic capability, should allow a firm to adapt its knowledge base to deal with changing environments (Wang and Ahmed, 2007). The ability to sense new business opportunities by understanding how newly acquired knowledge can be integrated or adapted to the existing technological resources and capabilities are path-dependent of previous learning processes. Deliberated and experience-based learning investments are needed in order to develop such abilities (Zollo and Winter, 2002) and convert them into a source of competitive advantage, as they are valuable and inimitable (Barney, 1991).

From these arguments, this paper suggests that new knowledge from company members derived from internal SC can increase in value if its assimilation and integration into the firm’s common knowledge base is properly done. In that case, this kind of knowledge could also become a source of new innovative results. Consequently, when a firm has not properly developed these abilities, the achievement of benefits from internal SC is limited (Yu, 2013). For this reason, a company’s capabilities to absorb and exploit knowledge will mediate the relationship between intra-organizational SC and its product innovation capabilities. This second hypothesis is formulated as follows:

H_2_: A company’s RACAP will have a mediating effect on the relationship between intra-organizational SC and product innovation capabilities.

## Sample and Methodology

The empirical analysis was carried out based on a sample of Spanish companies from innovation-intensive industries such as biotechnology and the pharmaceutical industry, where RACAP is an essential capability. To collect company data and information, the SABI (a system for accounting information analysis in Spanish and Portuguese firms) database was used. As the search we used a criterion the Spanish industry classification CNAE-2009, achieving a population of 735 firms. Consequently, an on-line survey was designed and launched, including questions relating to innovation, absorptive capacity and SC. As a previous step to launching the survey, a pre-test was conducted in order to analyze its reliability^2^.

For the measurement of the research variables, we adapted Likert scales from 1 to 7, which other studies have previously used and validated (see Appendix for the list of items). Measures for the variables of the study included the following: (1) eight items about the firm’s realized absorptive capacity (R_AC) according to Jansen et al. (2005); (2) 14 items representing intra-organizational SC (INT_SC)^3^ according to Tsai and Ghoshal (1998), Maurer et al. (2011), Máynez et al. (2012), Cuevas et al. (2014), and Horn et al. (2014); and (3) five items reflecting product innovation (PROD_INN) based on Škerlavaj et al. (2010). The research specifications are included in Table 1.

**TABLE 1:** Research specifications.

Population	735
Geographical scope	Spain
Sample size	87 firms
Unit of analysis	Firm or business unit
Data collection method	Online survey
Response rate	11.84%
Sampling error	9.87%; *p* = *q* = 0.5
Confidence level	95%
Type of sampling	Convenience

Finally, the Harman Test^4^ was applied to evaluate if the existence of common variance could be a concern for the collected set of data. This analysis confirms our study’s data validity.

## Statistical Analysis and Results

For testing the hypotheses, a structural equation model (SEM) using the partial least squares (PLS) technique and SmartPLS 3.2. software was applied. PLS is a multivariate analysis technique (Wold, 1985), based on variance analysis, used to model latent constructs under non-normality conditions for data and small sample sizes (Hair et al., 2013), which is typically applied in two stages:

### Measurement Model

Confirmatory factor analysis was used to estimate the measurement model in order to assess the reliability (individual items and constructs) and convergent and discriminant validity of measures. The findings (Table 2) corroborate the reliability and validity of the measurement model.

**TABLE 2:** Measurement model.

Constructs	Range of loadings	CR	AVE	Correlations							
				
				INT_SC	INT_SSC	INT_RSC	INT_CSC	R_AC	ASIM/TRANSF	EXPLOT	PROD_INN
INT_SC (2nd order)		0.925	0.805	0.897							
INT_SSC	0.753–0.908	0.923	0.707		0.841*						
INT_RSC	0.939–0.965	0.976	0.909		0.364	0.954*					
INT_CSC	0.786–0.918	0.923	0.707		0.791	0.705	0.867*				
R_AC (2nd order)		0.827	0.708					0.841			
ASIM/TRANSF	0.722–0.865	0.917	0.689		0.591	0.611	0.715		0.830*		
EXPLOT	0.714–0.835	0.809	0.587		0.283	0.267	0.366		0.456	0.766*	
PROD_INN	0.709–0.853	0.868	0.569		0.410	0.364	0.506		0.580	0.311	0.755

*Root of the variance shared between the first- order constructs and their measures. Diagonal elements (in italics) are the square root of the variance shared between the constructs and their measures; off-diagonal elements are the correlations between the items/constructs; for discriminant validity, diagonal elements should be higher than off-diagonal elements in the same row and column (Fornell and Larcker, 1981).

Individual item reliability was assessed using standardized loadings (λ), which is acceptable when the value is at least 0.707 (Chin, 1998; Hair et al., 2013). Moreover, construct reliability was analyzed by the Composite Reliability Index (CRI). The CRI must be at least 0.7 in early research stages and reach a stricter value of 0.8 for more advanced research stages (Nunnally, 1978).

Convergent validity is analyzed by examining the average variance extracted (AVE), which should be higher than 0.5 as a minimum value (Fornell and Larcker, 1981). Finally, discriminant validity confirms the extent of a construct being structurally different from other constructs. For that purpose, the AVE for each construct should be higher than the variance that such a construct shares with the rest of the model constructs (Fornell and Larcker, 1981).

### Structural Model

**FIGURE 1:**
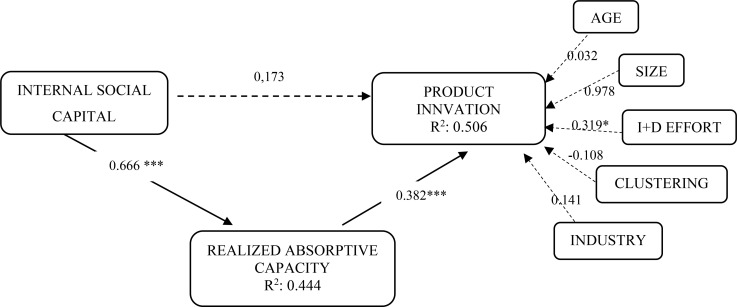
Research model and results. *p < 0.05 (*t*_(0.05; 4999)_ = 1.6479); ***p < 0.001 (*t*_(0.001; 4999)_ = 3.1066).

The structural model analysis is applied for testing the proposed hypotheses by analyzing both path coefficients (β) and determination coefficients (R^2^) (Figure 1). Specifically, for the examination of the direct influence of intra-organizational SC on RACAP (H_1_), the relationship between both variables is positive and significant (β = 0.666 *p* < 0.001). As a result, this first hypothesis is supported (Table 3).

**TABLE 3:** Hypothesis 1 test.

Effect on dependent variable	*Path* coefficient (β)	*t*	Confidence interval 95%
INT_SC → R_AC	0.666***	9.998	0.528–0.789

***p < 0.001 (*t*_(0.001; 4999)_ = 3.1066).

Partial least squares uses bootstrapping for testing mediating effects, providing indicators for both direct and indirect effects (Hayes and Scharkow, 2013). According to Nitzl et al. (2016), the results must comply with four conditions (Table 4). Specifically, for the mediating effect proposed by H_2_, the indirect effect between intra-organizational SC and product innovation when the mediating variable RACAP is introduced in the research model is strong and highly significant (β = 0.255, *p* < 0.001). However, the direct path coefficient in the relationship between internal SC and product innovation is not significant when the mediating variable is introduced into the research model (β = 0.178, *p* > 0.05). Consequently, there is a total mediating effect of RACAP on the relationship between intra-organizational SC and product innovation. In order to complement this analysis, a percentile approach was applied, for both direct and indirect effects. The results confirm that only the relationship between intra-organizational SC and product innovation shows a confidence interval that contains the zero value when the mediating variable is introduced into the research model, remaining significant for all the other effects (Chin, 2010).

**TABLE 4:** Mediating hypothesis 2 test.

Effect on dependent variable INT_SC → PROD_INN	*Path* coefficient (β)	t	Confidence interval 95%
Direct	0.178	1.278	−0.101 to 0.423
Indirect	0.255***	3.149	0.118 to 0.425

***p < 0.001 (*t*_(0.001; 4999)_ = 3.1066).

Furthermore, R^2^ coefficients indicate the amount of variance explained by the relationships in the model. Figure 1 shows that the model explains 50.6% of the variance of product innovation and 44.4% of the variance of RACAP. Authors such as Falk and Miller (1992) suggest that this value should be at least 10% for a model to be considered as having enough predictive power, a condition fulfilled by our study model.

Finally, regarding control variables, only R&D effort has a significant effect on product innovation (β = 0.319, *p* < 0.05). This is a logical result if we consider that those firms that make higher innovation efforts achieve an increase in their ability to create new products.

## Discussion and Conclusion

This study has shown the existing relationships between intra-organizational SC, RACAP and product innovation capabilities in a sample of firms in the Spanish biotechnological industry. The testing of the model shows the positive impacts of, on the one hand, internal SC (a construct that includes structural, relational and cognitive SC) and RACAP (hypothesis 1) and, on the other hand, RACAP and a company’s product innovation capabilities. As explained in our theoretical background section, innovation capabilities have been shown to be a positive result of the development of RACAP in different contexts (Ebers and Maurer, 2014; Leal-Rodríguez et al., 2014; Flor et al., 2018). A firm that is capable of integrating transforming and adjusting external knowledge to its existing knowledge base will have further opportunities to learn how to develop innovative activities such as new product development (Cohen and Levinthal, 1989; Schilling, 2019).

Regarding the first hypothesis, the obtained results support the idea that firmly established and frequent links between a company’s employees, trusting relationships and the development of common codes to interact with one another lead a firm to improve its capacity to integrate/transform and use knowledge. Maurer et al. (2011) used the concept of knowledge transfer processes in order to give an explanation regarding the positive relationship between intra-organizational relationships and dynamic capabilities to exploit external knowledge. Increasing interactions between employees (coupled with knowledge transfer) give the firm the opportunity to identify where external resources and how newly acquired knowledge should be integrated into other existing resources when personal knowledge is highly disseminated in a firm. In fact, as RACAP is a function of patterns of learning in a firm, the more a firm’s employees try to seek out peers and interaction elsewhere to solve problems, the more the company learns to find solutions based on knowledge exploitation over time.

At this point, it is also important to differentiate between PACAP and RACAP and the role they play regarding knowledge acquisition and its exploitation by firms. While PACAP is closely connected with knowledge identification and assimilation, and inter-organizational relationships are thus essential aspects for detecting sources to acquire knowledge, RACAP is more dependent on internal processes to learn how to exploit the acquired knowledge (Ortiz et al., 2018). These internally learned processes are guided by the way a company is able to manage employees’ interactions to transfer tacit and codified knowledge by means of networks based on trust and shared understandings about behaviors, the functioning of activities and its competitive objectives.

On the whole, internal SC is likely to create routines for transforming and using new knowledge as employees will be able, owing to the presence of personal and company networks, trust and common norms, to test newly acquired technology with trusted colleagues, and also eschew knowledge which has no practical use for the running of company activities. Moreover, internal SC would have a positive effect on the organization’s ability to mitigate the potential confusion from the knowledge that a firm obtains from internal networks, improving KM internally (Jensen and Szulanski, 2007).

Moreover, the results of the study show that RACAP can be seen as a way to channel internal SC toward innovation. While most of the SC literature predicts a positive, direct impact of SC on product innovation (see e.g., Zheng, 2010; Yu, 2013; Xie et al., 2018), this paper shows that the empirical results of these studies may not be conclusive, as ideas and knowledge resulting from internal interactions should be previously integrated and exploited through dynamic capabilities for sensing and seizing new opportunities in a constantly changing environment (Teece et al., 1997). We thus propose a mediating relationship based on the absorptive capacity to understand the connection between internal SC and innovation. An important result of our study is the *total* mediating effect that has been found in our tested model by considering RACAP as the intermediate step between intra-organizational relationships and product innovation. This provides insights regarding the predecessors of RACAP, which is still considered a “black box” regarding competitive advantages based on innovation (Peeters et al., 2014), especially for the difficulty it poses to company management in practice. The total mediating effect means that when RACAP is introduced into the empirical model, the direct relationship between internal SC and product innovation ceases to be significant, meaning that internal SC without RACAP does not lead to knowledge resources based on networks achieving an improvement in innovation capabilities. From a managerial viewpoint, this result has an important implication: a firm should be aware that the promotion of intra-organizational relationships by means of developing shared values for knowledge exchange, and further (and firmly established) trustworthy links between employees should be connected to R&D efforts and the constant scanning for new opportunities to gain further innovation.

A theoretical implication for KM and SC literature comes from the confirmation of the total mediating effect in the second hypothesis. Hence, this paper has shown that RACAP allows firms to reconfigure and renew their knowledge base following a specific strategic direction (Wang and Ahmed, 2007). For a company, this would mean that its knowledge base is built over time and subject to path dependencies, and therefore not easily imitated by competitors (Barney, 1991). The abilities relating to the understanding of new opportunities through the identification and valuation of the firm’s internal technologies along with the understanding of how these technologies can interact with externally acquired knowledge shape the fundamentals of RACAP. Deliberated and experience-based learning investments would be needed in order to develop such abilities (Zollo and Winter, 2002). Moreover, a firm’s strategic focus will have an important influence on such development. This means that different strategies (e.g., cost leadership, differentiation) would influence a firm’s position with respect to knowledge exploitation, exploration or both (Wang and Ahmed, 2007). Although internal SC has been analyzed as an antecedent of RACAP in hypothesis one, a firm’s strategic focus is an aspect that has not been explicitly contemplated by our model. Future papers could take into consideration this relationship as an interesting line of research.

The study results have interesting prescriptive implications for company managers in high-tech industries such as biotechnology. First, managers should understand that “good” management of intra-organizational SC allows their companies to develop dynamic capabilities related to the exploitation of unique and complex knowledge. The ultimate goal is to expand, reconfigure and adapt their resources in order to deal with environmental change (Teece et al., 1997; Eisenhardt and Martin, 2000). Moreover, the development of cohesive links, along with common values and clear rules of exchange regarding internal knowledge should be oriented to improve their RACAP. By doing so, organizations could optimize their knowledge exploitation processes by selecting the best method(s) for integrating external and internal knowledge depending on their needs, timing, and particular circumstances (e.g., strategy). Furthermore, the study of the existing relationships between SC dimensions leads to a better understanding of their internal functioning and configuration, which constitutes an important issue for managers, who should consider not only the relevance of each type of SC for knowledge integration but also the value added that arises from their interdependencies.

An additional managerial implication of this paper is that it is necessary to develop strong and frequent links between employees in firms (structural internal SC) but it is also essential to create and develop cognitive SC (e.g., common rules; shared language) to take advantage of innovation via RACAP. The way in which firms develop and improve these social norms and mindsets has not been the focus of this paper, but it could be an interesting avenue for future research (e.g., how network agents should interact; how they should manage such processes).

Among the limitations of this study, we include, firstly, the cross-sectional nature of the empirical analysis. Furthermore, the study does not consider if there are dependent relationships between the structural, relational and cognitive dimensions of inter-organizational SC and RACAP, neither their influence on product innovation capability. Future studies may focus on such analysis. Additionally, we used self-reporting data. Despite the applied Harman test not showing this issue to be of significant concern, problems of common method variance could be present. Finally, we focus on the high knowledge-intensive industries to test our hypotheses, which might restrict the generalizability of the findings to additional industries or sectors with different features. To address this limitation, the study could be replicated for validation purposes in other contexts (low-tech industries; other countries). Also, a longitudinal study could be carried out, focusing on the analysis of how network and absorptive capacity configuration and relationships change over time and the influence of this on innovation performance.

Overall, internal social interactions are shown as mechanisms that allow people to learn how to share important information with each other, create a common understanding related to tasks or goals, as well as obtain other resources and ideas (Xie et al., 2018), in order to generate innovation via knowledge integration with other assets and their transformation. The generation and application of new ideas will therefore be promoted by social interaction, or in other words, the generation and application of new ideas to achieve further innovation will be promoted by a firm’s inter/organizational SC.

## Data Availability Statement

The raw data supporting the conclusions of this article will be made available by the authors, without undue reservation.

## Ethics Statement

Ethical review and approval was not required for the study on human participants in accordance with the local legislation and institutional requirements. Written informed consent for participation was not required for this study in accordance with the national legislation and the institutional requirements.

## Author Contributions

FG wrote the Introduction. BO developed the research model, the theoretical background, and fieldwork and empirical analysis. MD contributed with Discussion and Conclusion sections. However, there was continuous feedback among the authors over all research period. All authors contributed to the article and approved the submitted version.

## Conflict of Interest

The authors declare that the research was conducted in the absence of any commercial or financial relationships that could be construed as a potential conflict of interest.

## Footnotes

1 Product innovation has especially attracted interest from a number of organizational researchers as a result of absorptive capacity deployment when high-technology intensive industries are considered (Wang and Ahmed, 2007; Donate and Guadamillas, 2010).

2 Several scholars with an extensive publication record in KM and/or intellectual capital from the Business Department of Castilla-La Mancha University reviewed a draft of the questionnaire. Following this, various in-depth interviews with the CEOs of two biotechnological companies were carried out. Subsequently, the authors deleted or changed those items that seemed to be difficult to respond to or to understand.

3 Five items corresponding to structural social capital, four items to relational social capital and five items regarding cognitive social capital.

4 An exploratory factorial analysis (principal components with varimax rotation) was implemented. The results reveal four factors with eigenvalues above one, explaining 67.5% of the total variance. Because the first factor explains only 37.4% percent of the total variance, common variance does not appear to be a major problem in our research (Podsakoff and Organ, 1986).

## Appendix

**Table:** Appendix Research Items.

Constr.	Dimensions	My firm usually… (From 1 –strongly disagree to 7-strongly agree)
INTERNAL SOCIAL CAPITAL (INT_SC)	STRUCTURAL SOCIAL CAPITAL (INT_SSC)	Has employees who are very close to each other
		Has employees with a high level of communication with each other
		Has employees who enjoy spending time together
		Encourages its employees to have frequent contacts through, formal (e.g., corporate directory, meetings) and informal means (e.g., outdoor activities)
		(In general) Has employees who maintain relationships with each other
	
	RELATIONAL SOCIAL CAPITAL (INT_RSC)	(In general) Has employees with good intentions
		Has honest and trusted employees
		Has faultless employees
		Has totally confidence in its employees
	
	COGNITIVE SOCIAL CAPITAL (INT_CSC)	Has employees who are aware that pursuing a common organizational objective and mission is essential
		Fosters teamwork
		Fosters the open discussion of problems
		Fosters abilities such as creativity and flexibility
		Provides our employees with resources and time for learning and sharing.

REALIZED ABSORPTIVE CAPACITY (R_AC)	ASSIMILATION/TRANSFORMATION	Considers the consequences of changes in the market in order to create new products and services
		Uses ICT for registration and storage of new knowledge for future reference
		Recognizes the usefulness of new external knowledge and of incorporating it with existing knowledge quickly
		Establishes regular meetings to discuss the consequences of market trends and the development of new services
		Has tools/techniques for distributing and sharing knowledge
	
	EXPLOITATION	Studies which is the best way to exploit knowledge
		Knows which area (department, employees) can best exploit new knowledge
		Hardly ever uses new knowledge in new products*

PRODUCT INNOVATION (PROD_INN)		Has introduced more innovative products/services compared to its competitors
		Has frequently emphasized the development of new patented products
		Has satisfied the market through the speedy development of its products
		Has continuously changed product design to enter more quickly in new emerging markets
		Has continuously improved the components and quality of its products

**Item with inverse coding.*
